# Supplementary material for: High-speed volumetric two-photon fluorescence imaging of neurovascular dynamics
Source: Nat Commun. 2020 Nov 26;11:6020. doi: 10.1038/s41467-020-19851-1 (PMC7693336; doi:10.1038/s41467-020-19851-1)
Supplement: Supplementary file 1 — Supplementary Information [file 41467_2020_19851_MOESM1_ESM.pdf]

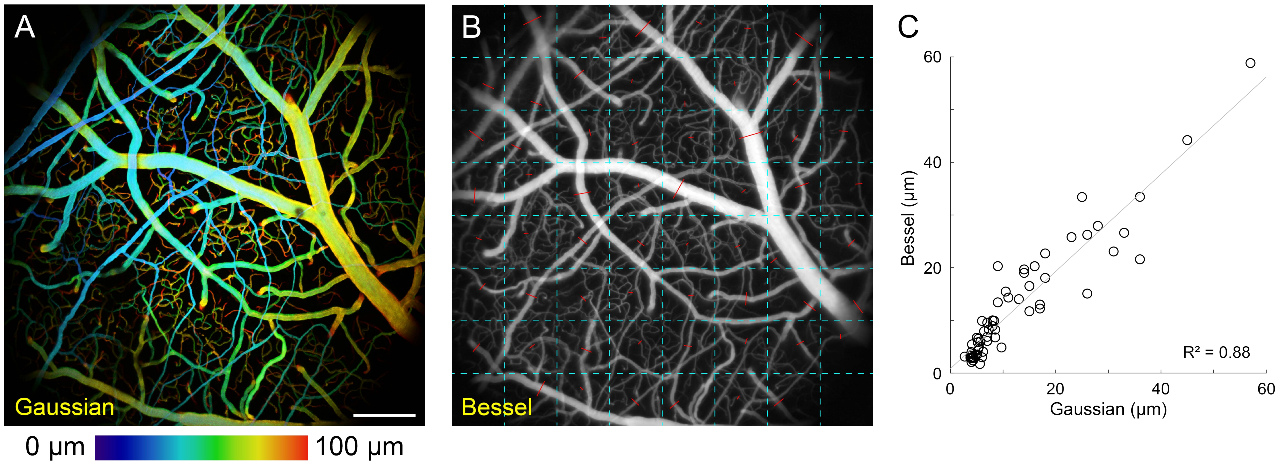

Supplementary Figure 1. Relationship between blood vessel diameters measured with Gaussian versus Bessel TPLSM methods.

(A) Gaussian image stack of a 1.4 mm x 1.4 mm x 100 µm volume of vasculature, color coded by depth. (B) Bessel image of the same volume in A, visualized in grayscale on the normalized square root of fluorescence signal and divided into 8 x 8 equal subregions (cyan dashed lines). One vessel segment was chosen within each subregion except the 4 corner subregions (60 total). Red lines indicate the line along which fluorescence signal and vessel diameter were determined from Gaussian and Bessel image data. (C) Scatter plot of the same vessel diameters measured from Gaussian and Bessel data captured at different times. Scale bar: 200 µm. Representative data from 4 mice. Post-objective power: Gaussian: 45 mW; Bessel: 217 mW.

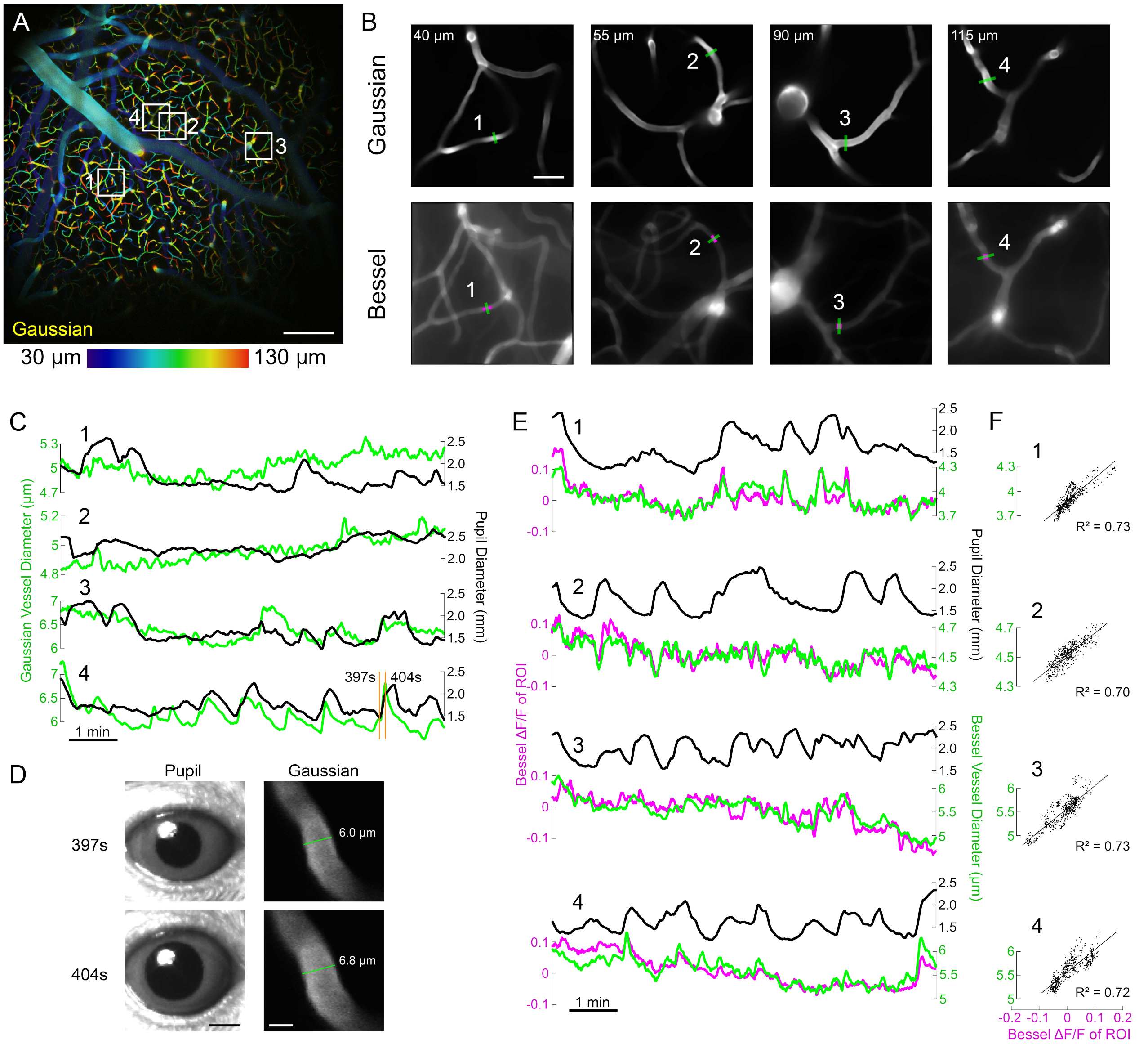

Supplementary Figure 2. Capillary dilation and constriction probed by Gaussian and Bessel TPLSM.

(A) A 1.4 mm x 1.4 mm x 0.1 mm volume of vasculature imaged with Gaussian TPLSM, color coded by depth. (B) High-resolution Gaussian and Bessel images of capillaries. For each area (within the white squares in A), Gaussian images were taken first, followed by Bessel images, both acquired at 30 Hz with 0.2 µm pixel size for 8 minutes. (C) Time traces of diameters of 4 capillary segments from Gaussian images in B, plotted with simultaneously measured pupil diameter. (D) Example pupil and 1-second averaged Gaussian images of Capillary 4 at two time points (indicated by yellow lines in C) showing changes in pupil and capillary diameters. (E) Time traces of fluorescence signal changes and diameters of the same 4 capillary segments from Bessel images in B, plotted with simultaneously measured pupil diameter. Unlike larger vessels whose size correlations with pupil diameter were time-invariant (Fig. 4D), capillaries had time-varying size correlations with pupil diameter over minutes. (F) Scatter plot of fluorescence versus capillary diameter data in E. Scale bars: A: 200 µm, B: 20 µm, D: Pupil: 1mm, Gaussian: 5 µm. Representative data from 2 mice. Post-objective power: Gaussian: 17 mW, Bessel: 112 mW.

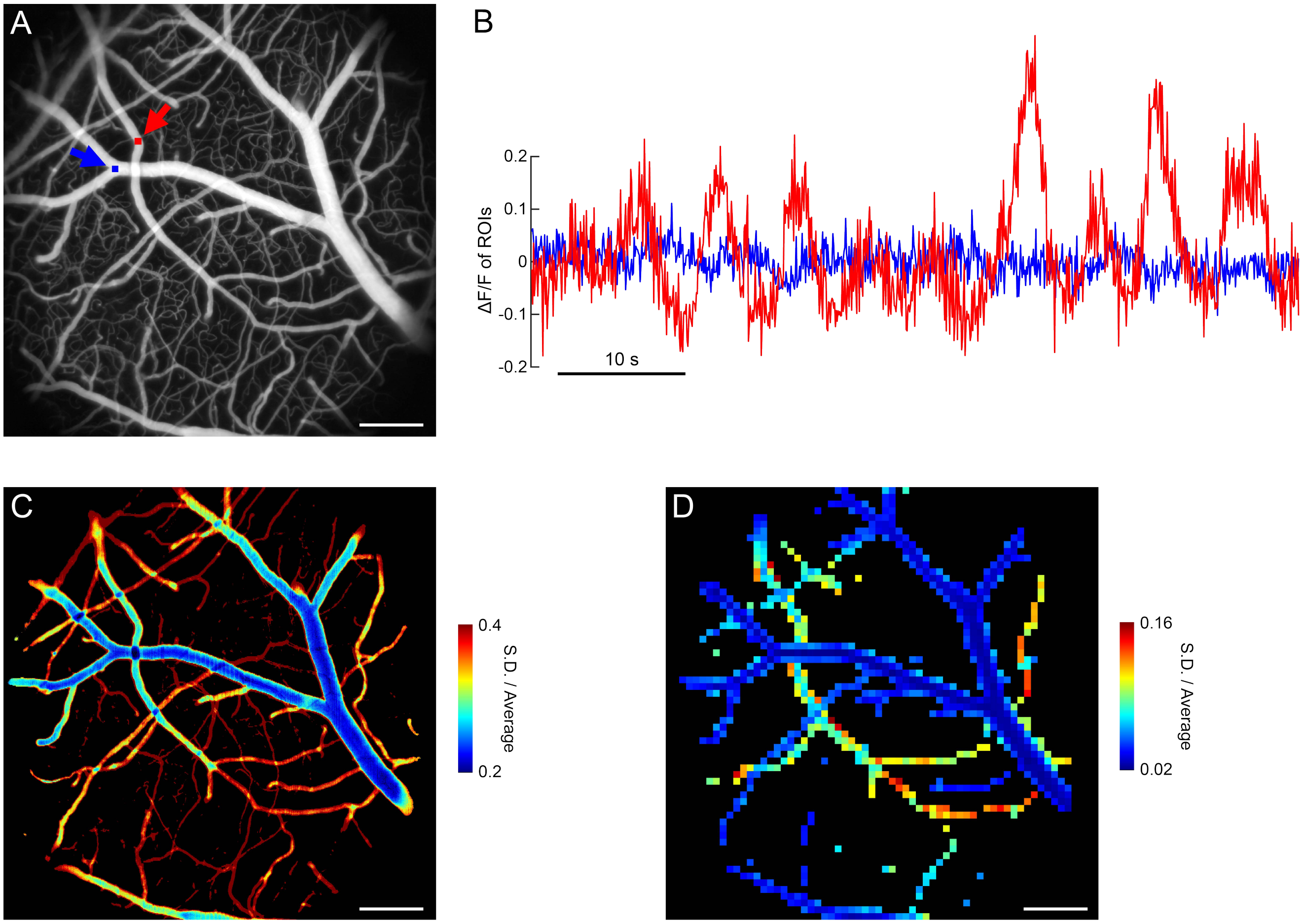

Supplementary Figure 3. Arterioles exhibited greater changes in Bessel fluorescence signal than venules.

(A) Bessel TPLSM image with the ROIs from Fig. 3F (blue square and arrow) and Fig. 3G (red square and arrow) shown. (B) Fluorescence time traces of the ROIs from A showing a large difference in their magnitudes of fluorescence signal change. (C) Standard deviation (S.D.) divided by average signal for the brightest 20% of pixels in A (representing vasculature). The dimmest 80% of pixels (representing non-vasculature tissue) are represented in black. (D) Standard deviation divided by average signal for the brightest 20% of all ROIs from Fig. 3F, G. Both C and D show a clear distinction between arteriole and venule populations. Representative data from 4 mice. Scale bars: 200μm for A, C, D.
