# Supplementary material for: High-speed volumetric two-photon fluorescence imaging of neurovascular dynamics
Source: Nat Commun. 2020 Nov 26;11:6020. doi: 10.1038/s41467-020-19851-1 (PMC7693336; doi:10.1038/s41467-020-19851-1)
Supplement: Supplementary file 3 — Description of Additional Supplementary Files [file 41467_2020_19851_MOESM3_ESM.pdf]

Description of Additional Supplementary Files

File Name: Supplementary Movie 1
Description: **Video summary of *in vivo* structural imaging of vasculature using Gaussian and Bessel focus scanning.**

Individual Gaussian frames at different depths are followed by the color-coded Gaussian stack. Then, Bessel image (100-frame average) of the same 1.4 mm × 1.4 mm × 110 µm volume is shown followed by individual Bessel frames imaged at 15 Hz. All grayscale images are plotted using the normalized linear fluorescence signal.

File Name: Supplementary Movie 2
Description: **Bessel TPLSM imaging of vasodilation and vasoconstriction in 3D.**

1-minute-long Bessel TPLSM images of a 1.4 mm x 1.4 mm x 0.1 mm vasculature volume imaged for 1 minute, showing vasodilation and vasoconstriction of 3D vasculature. Inset shows a zoomed-in video of a vessel segment (same as the inset in Fig. 3A). Each frame in the video is the 5-frame-average of the 15 Hz data. Images are plotted in grayscale using the normalized linear fluorescence signal.

File Name: Supplementary Movie 3
Description: **Bessel TPLSM imaging of a 3D vasculature network reveals entrainment of vasodilation and vasoconstriction with pupil diameter.**

10-minute-long Bessel TPLSM recording of a 1.4 mm x 1.4 mm x 0.1 mm volume vasculature at 15 Hz, with zoomed-in insets of two vessel segments (ROI1 and ROI2) and the concurrently recorded mouse pupil images. Vessels in ROI 1 and ROI 2 show anticorrelated vasodilation and vasoconstriction dynamics and correlation with pupil size. Each frame in the video is the 15-frame-average of the 15 Hz data. Images are plotted in grayscale using the normalized linear fluorescence signal.

File Name: Supplementary Movie 4
Description: **High-speed volumetric measurement of cerebral blood flow speed with Bessel TPLSM.**

5-second-long Bessel TPLSM recording of blood flow in a 416 µm x 416 µm x 80 µm volume vasculature at 99 Hz. Images are plotted in grayscale using the normalized linear fluorescence signal.
