# Supplementary material for: High-speed volumetric two-photon fluorescence imaging of neurovascular dynamics
Source: Nat Commun. 2020 Nov 26;11:6020. doi: 10.1038/s41467-020-19851-1 (PMC7693336; doi:10.1038/s41467-020-19851-1)
Supplement: Supplementary file 8 — Reporting Summary [file 41467_2020_19851_MOESM8_ESM.pdf]

nature research

Corresponding author(s): Na Ji

Last updated by author(s): Sep 29, 2020

Reporting Summary

Nature Research wishes to improve the reproducibility of the work that we publish. This form provides structure for consistency and transparency in reporting. For further information on Nature Research policies, see our Editorial Policies and the Editorial Policy Checklist.

Statistics

For all statistical analyses, confirm that the following items are present in the figure legend, table legend, main text, or Methods section.

n/a	Confirmed	
☐	☒	The exact sample size (n) for each experimental group/condition, given as a discrete number and unit of measurement
☐	☒	A statement on whether measurements were taken from distinct samples or whether the same sample was measured repeatedly
☒	☐	The statistical test(s) used AND whether they are one- or two-sided<br>*Only common tests should be described solely by name; describe more complex techniques in the Methods section.*
☒	☐	A description of all covariates tested
☒	☐	A description of any assumptions or corrections, such as tests of normality and adjustment for multiple comparisons
☐	☒	A full description of the statistical parameters including central tendency (e.g. means) or other basic estimates (e.g. regression coefficient) AND variation (e.g. standard deviation) or associated estimates of uncertainty (e.g. confidence intervals)
☒	☐	For null hypothesis testing, the test statistic (e.g. F, t, r) with confidence intervals, effect sizes, degrees of freedom and P value noted<br>*Give P values as exact values whenever suitable.*
☒	☐	For Bayesian analysis, information on the choice of priors and Markov chain Monte Carlo settings
☒	☐	For hierarchical and complex designs, identification of the appropriate level for tests and full reporting of outcomes
☒	☐	Estimates of effect sizes (e.g. Cohen's d, Pearson's r), indicating how they were calculated

Our web collection on statistics for biologists contains articles on many of the points above.

Software and code

Policy information about availability of computer code

Data collection	2-Photon image acquisition was performed on the ThorImage v3, v4 program. Pupil image acquisition was performed using a MATLAB 2019 script. Data collection methods used in this manuscript are not specific to the programs used, and can work with other image acquisition software.
Data analysis	Fiji (ImageJ 1.53c), MATLAB 2019.<br>Custom skeletonization, blood vessel tracing, and pupil correlation code in MATLAB can be accessed at: https://figshare.com/projects/Bessel_Vessel/89135

For manuscripts utilizing custom algorithms or software that are central to the research but not yet described in published literature, software must be made available to editors and reviewers. We strongly encourage code deposition in a community repository (e.g. GitHub). See the Nature Research guidelines for submitting code & software for further information.

Data

Policy information about availability of data

All manuscripts must include a data availability statement. This statement should provide the following information, where applicable:

- Accession codes, unique identifiers, or web links for publicly available datasets
- A list of figures that have associated raw data
- A description of any restrictions on data availability

Raw image sequences used to generate Figures 2, 3, 5, and Supplementary Figures 1, 3 are available on https://figshare.com/projects/Bessel_Vessel/89135 . Raw data used to generate Figure 4 and Supplementary Figure 2 have large file sizes (> 5 GB) and are available upon reasonable request to the corresponding author.

Field-specific reporting

Please select the one below that is the best fit for your research. If you are not sure, read the appropriate sections before making your selection.

☒ Life sciences ☐ Behavioural & social sciences ☐ Ecological, evolutionary & environmental sciences

For a reference copy of the document with all sections, see nature.com/documents/nr-reporting-summary-flat.pdf

Life sciences study design

All studies must disclose on these points even when the disclosure is negative.

Sample size	7 mice were imaged for all results in this manuscript. No sample size calculation was performed. The number of mice were chosen to sufficiently demonstrate our imaging technology and application.
Data exclusions	No two-photon data was excluded. Pupil data was excluded if the animal was excessively squinting or closing its eye.
Replication	Similar results have been obtained over multiple experiments, with sample sizes reported in the paper. All replication attempts were successful.
Randomization	No randomization was incorporated into this study. Randomization was not relevant to this study because we are not comparing between samples.
Blinding	Blinding was not applicable in our data acquisition and analysis because we do not make comparisons between samples or populations. Raw images indicated well-isolated blood vessels and analyses were primarily carried out in blood vessels with high signal to background.

Reporting for specific materials, systems and methods

We require information from authors about some types of materials, experimental systems and methods used in many studies. Here, indicate whether each material, system or method listed is relevant to your study. If you are not sure if a list item applies to your research, read the appropriate section before selecting a response.

Materials & experimental systems

n/a	Involved in the study	
☒	☐	Antibodies
☒	☐	Eukaryotic cell lines
☒	☐	Palaeontology and archaeology
☐	☒	Animals and other organisms
☒	☐	Human research participants
☒	☐	Clinical data
☒	☐	Dual use research of concern

Methods

n/a	Involved in the study	
☒	☐	ChIP-seq
☒	☐	Flow cytometry
☒	☐	MRI-based neuroimaging

Animals and other organisms

Policy information about studies involving animals; ARRIVE guidelines recommended for reporting animal research

Laboratory animals	Wild-type (Jackson Laboratories, Black 6, stock no. 000664)<br>Thy1-GFP (neuronal green fluorescence protein expression, Jackson Laboratories, Tg(Thy1-EGFP)MJrs/J, stock no. 007788)<br>Aldh1l1-GFP (pan-glial green fluorescence protein expression, Mutant Mouse Resource & Research Centers, Tg(Aldh1l1-EGFP)OFC789Gsat/Mmucd, stock no. 011015-UCD)<br><br>All laboratory mice were 3-4 months old. Both female and male mice were used. Animals were housed socially in normal light cycle and room temperature.
Wild animals	The study did not involve wild animals.
Field-collected samples	The study did not involve field-collected samples
Ethics oversight	All animal experiments were conducted according to the National Institutes of Health guidelines for animal research. Procedures and protocols on mice were approved by the Animal Care and Use Committee at the University of California, Berkeley.

Note that full information on the approval of the study protocol must also be provided in the manuscript.
